# Broadband Coherent Raman Scattering: Excitation Architectures and Operating Regimes

**DOI:** 10.3390/molecules31071207

**Published:** 2026-04-06

**Authors:** Roland Ackermann, Timea Koch, Tom Lippoldt, Thomas Gabler, Stefan Nolte

**Affiliations:** 1 Abbe Center of Photonics, Institute of Applied Physics, Friedrich-Schiller-Universität Jena, Albert-Einstein-Straße 15, 07745 Jena, Germany; timea.koch@uni-jena.de (T.K.); tom.lippoldt@uni-jena.de (T.L.); thomas.gabler@uni-jena.de (T.G.); stefan.nolte@uni-jena.de (S.N.); 2 Fraunhofer Institute for Applied Optics and Precision Engineering IOF, Albert-Einstein-Straße 7, 07745 Jena, Germany

**Keywords:** Raman spectroscopy, few-cycle pulse, ultrabroadband pulse, hollow-core fiber, optical parametric chirped pulse amplification, photonic crystal fiber

## Abstract

Coherent Raman scattering (CRS) techniques such as coherent anti-Stokes Raman scattering (CARS) provide chemically specific vibrational contrast with signal levels far exceeding spontaneous Raman scattering (SpRS). Extending these to broadband excitation enables multiplex detection across wide spectral regions, including the fingerprint region, CH-stretch bands and high-frequency vibrational modes. This review provides a structured overview of excitation architecture for broadband CRS, ranging from low-energy oscillator schemes to energy-scalable platforms. The discussion is organized along key design parameters, including spectral bandwidth, excitation intensity, and probe delay, which jointly determine the accessible operating regimes. Rather than representing competing methods, the reviewed architectures are presented as a complementary toolbox for application-driven spectroscopy in chemically reactive environments and complex biological systems. In addition, a representative OPCPA-based implementation is presented as a platform demonstration to illustrate accessible operating regimes, single-shot stability, and multiplex detection capability under realistic experimental conditions.

## 1. Introduction

Vibrational spectroscopy provides chemically specific insight into molecular structure and composition. Among nonlinear implementations, coherent Raman scattering (CRS) techniques based on third-order nonlinear susceptibilities have emerged as powerful tools for label-free chemical detection. CRS encompasses approaches such as coherent anti-Stokes Raman scattering (CARS), coherent Stokes Raman scattering (CSRS) and related four-wave-mixing schemes that probe molecular vibrations. In the following, we use CRS as the umbrella term and refer to specific implementations where appropriate.

In practical implementations, CARS has become the most widely adopted CRS modality. An advantage of CARS is that the anti-Stokes signal is blue-shifted relative to the excitation fields, facilitating spectral separation from pump and Stokes beams and often reducing interference from fluorescence background. However, non-resonant background contribution remains intrinsic to CRS processes and must be addressed separately. In practice, many broadband excitation architectures and detection strategies have been developed primarily within the CARS framework. The underlying considerations, however, extend across coherent Raman modalities.

CARS was originally developed as a diagnostic tool for combustion and gas-phase thermometry, where its high signal strength allowed non-intrusive measurements of temperature and species concentration measurements in flames and reacting flows [1,2]. In this original approach, high signal strength comes at the expense of requiring the Raman transition to be fixed and known in advance. As a nonlinear χ^(3)^-process, CARS involves four electromagnetic waves, which require phase-matching for efficient signal generation. To obey this condition, the established method is BOXCARS, where phase-matching is fulfilled by choosing proper focusing angles for the fixed pump, Stokes and probe wavelengths [3].

Broadband CARS and related multiplex schemes go beyond single-transition measurements by providing Raman-like spectra over large spectral widths—ideally from the fingerprint region (~300–1900 cm^−1^), extending through the OH/CH-stretch bands (2850–3750 cm^−1^) and up to high-frequency vibrations such as the H-H stretch in H_2_ (~4200 cm^−1^). The ability to access extended spectral regions in a single acquisition fundamentally expands the information content of CRS measurements. Broadband excitation enables (i) multiplex detection, i.e., the simultaneous observation of multiple Raman bands within a single acquisition without spectral scanning. Importantly, multiplex detection does not necessarily require ultrabroadband bandwidth but already applies to the simultaneous detection of several vibrational features within a limited spectral window, as demonstrated in early CARS studies (e.g., $\Delta \tilde{\nu }$ ~ 300 cm^−1^ [4]). Increasing bandwidth extends this concept by enlarging the accessible Raman shift range and thereby increasing the potential number of observable species and transitions. The effective multiplex capacity, however, is ultimately limited by spectral resolution, SNR, and NRB contributions. In particular, the spectral resolution—determined by the probe bandwidth and detection system—defines the minimum separation of distinguishable Raman features and thus the number of simultaneously resolvable bands. Beyond multiplex detection, broadband excitation enables (ii) temperature retrieval in gas-phase diagnostics via analysis of ro-vibrational population distributions, and (iii) structural discrimination in biomedical and chemical imaging through combined access to the fingerprint and the CH-stretch regions.

As mentioned above, conventional three-beam CARS relies on carefully chosen beam crossing angles. This concept becomes less straightforward in broadband excitation schemes. The simultaneous presence of a wide range of frequency components leads to a distribution of phase-mismatch conditions that cannot be satisfied simultaneously. In low-dispersion environments such as gases, quasi-collinear configurations can still provide efficient signal generation over extended bandwidth [5]. In contrast, condensed media such as liquids or biological tissue exhibit significantly stronger dispersion, resulting in larger phase mismatch across the spectral range and thus more stringent phase-matching constraints.

A key strategy to overcome this limitation is tight focusing. As shown by Bjorklund [6], the effective interaction length in a focused beam is reduced to the Rayleigh length range, thereby relaxing phase-matching conditions. In practice, this implies that broadband CARS excitation benefits from sufficiently large NA. Accordingly, modern CARS microscopy implementations would optimally employ high-NA objectives (e.g., NA ~ 0.8–1.2 [7,8]). From the underlying phase-matching considerations, this can be interpreted as requiring minimal apertures on the order of NA ≳ 0.2–0.4 for efficient broadband excitation (e.g., [9]).

Pioneering work around the turn of the millennium was conducted by the Silberberg group with modestly short pulses of 20–30 fs from Ti:sapphire oscillators, limiting the bandwidth to ~800 cm^−1^ [10,11,12]. In the years that followed, the technique diversified into distinct applications domains, which most prominently are the high pulse energy community focusing on gas analysis, and the biomedical nonlinear microscopy community. While both rely on providing sufficient spectral bandwidth for multiplex detection, emphasis and challenges differ substantially.

Gas and remote sensing scenarios typically require high pulse energies to compensate for low molecular densities, or inefficient signal generation from geometrical constraints, e.g., in an industrial, high-pressure combustion device. This shifts the scientific and technical challenges toward the generation and delivery of broadband, high energy excitation pulses. They are often on a mJ–mJ scale, using optical parametric amplifiers (OPAs) or hollow-core fibers. By contrast, biological imaging must rely on low energy pulses to avoid sample damage. These pulses are easily delivered by fs-oscillators at tens of MHz, shifting the research focus toward efficient spectral encoding, fast detection and background management. This divergence has also led to differences in terminology, which can cause confusion where the two communities overlap. The biomedical community commonly uses the term BCARS to describe a range of excitation schemes [8], whereas the high pulse energy community tends to emphasize the specific implementation, e.g., ultrabroadband two-beam CARS [13], without an established umbrella acronym.

A general introduction to broadband CRS techniques and their historical evolution is available in established reviews, which cover the third-order formalism and typical implementations for microscopy [14]. Rather than revisiting these foundations, we concentrate here on the excitation architectures that ultimately determine accessible bandwidth, pulse energy (⪆1 mJ) and repetition rate, as well as the associated practical constraints.

As outlined above, broadband CRS has evolved along two partially distinct trajectories: high energy gas-phase diagnostics, where the central challenge lies in generating and delivering spectrally broad, intense excitation pulses, and biomedical or chemical imaging, where limitations in tissue-safe pulse energy shifts the emphasis toward spectral encoding, non-resonant background (NRB) removal, and high-speed detection.

To reflect this divergence, we decouple bandwidth generation from spectral encoding and detection and discuss broadband CRS approaches along these largely independent design axes. We begin with excitation strategies that allow for large spectral bandwidths at increasing pulse energy. In addition, we include a representative experimental implementation to illustrate how these design considerations translate into practical regimes, including accessible bandwidth, single-shot stability, and multiplex detection capability.

## 2. Bandwidth Generation in Broadband CRS

Broadband CRS is governed not merely by the available spectral bandwidth, but by how this bandwidth is structured to drive and probe molecular coherence. Two aspects are central: (i) the spectral configuration of the excitation fields and (ii) the temporal relation between excitation and probe pulses. In the spectral domain, the CARS field can be written as [15,16]

(1) $$E_{\mathrm{CARS}}=\int_{0}^{\infty }d\Omega E_{\mathrm{probe}}(\omega -\Omega )\chi^{\left(3\right)}(\Omega )A(\Omega ),$$

with

(2) $$A(\Omega )=\int_{0}^{\infty }d\omega^{\prime }|E_{\mathrm{pump}}(\omega^{\prime })E_{\mathrm{Stokes}}(\omega^{\prime }-\Omega )|\;e^{i\Delta \varphi }.$$

Here, χ^(3)^ denotes the third-order susceptibility of the medium, comprising both resonant and non-resonant contributions. A(Ω) represents the spectral driving term generated by the pump and Stokes fields. Importantly, A(Ω) depends not only on the available spectral bandwidth, but also on the relative spectral phase Δφ between the fields. Broadband excitation therefore constitutes a controlled interference process: both spectral amplitude and phase determine how vibrational coherence is generated and how resonant contributions interfere.

If the spectral bandwidth is too narrow, only a limited set of Raman shifts Ω can be driven efficiently, and distinct pump and Stokes components are required to generate vibrational coherence. In contrast, sufficiently broad spectra allow different frequency components within a single pulse to behave as an intrapulse interaction, relaxing the need for spectrally separated beams.

**Figure 1:**
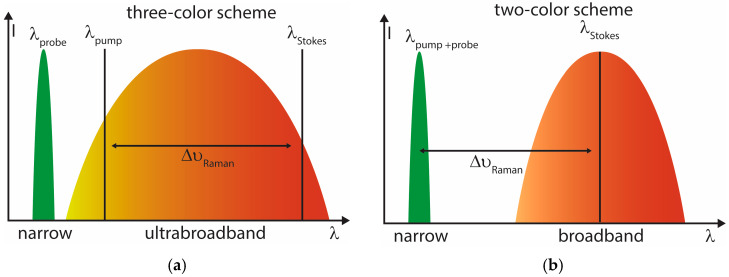
Schematic representation of (**a**) three-color and (**b**) two-color broadband CARS excitation. In the three-color scheme, vibrational coherence is generated by spectral components within an ultrabroadband pulse and read out by a separate narrowband probe. In the two-color scheme, a narrowband field serves as both pump and probe while broadband excitation determines the accessible Raman shifts. The color coding is used to qualitatively indicate the spectral position of the pulses.

A useful distinction therefore emerges between two-color and three-color excitation schemes (Figure 1). In the three-color scheme (Figure 1a), two distinct spectral components within an (ultra)broadband excitation pulse generate the intrapulse vibrational coherence, and a third field—typically narrowband—probes the coherence. In the two-color scheme (Figure 1b), a narrowband field acts as both pump and probe, while a second broadband field provides Stokes interaction. This classification depends solely on the excitation fields and is independent of probe timing.

While this classification is purely based on the spectral composition of the fields, it has practical implications for temporal operation. In two-color schemes, where pump and probe are typically derived from the same narrowband field, excitation and probing are often intrinsically linked in time, and independent delay control requires additional beam splitting and delay stages. In contrast, three-color schemes employ a separate probe field, allowing more direct and flexible adjustment of probe timing. However, delayed probing is not restricted to three-color configurations and is routinely implemented in conventional narrowband CARS (e.g., BOXCARS geometries), where pump and probe are spectrally identical but temporally separated.

**Figure 2:**
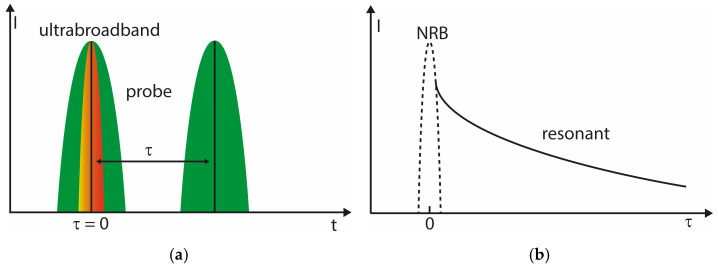
Temporal probing strategies and delay-dependent separation of resonant and non-resonant contributions in broadband CARS. (**a**) Schematic time-domain representation of ultrabroadband excitation and narrowband probe pulses. At zero delay (τ = 0), probe (green) and excitation fields overlap temporally. For τ > 0, the probe interrogates the vibrational coherence after excitation. (**b**) Qualitative dependance of signal intensity on probe delay τ. The NRB arises only during pulse overlap and decays essentially instantaneously, whereas the resonant contribution persists over the vibrational dephasing time. Delayed probing can therefore suppress NRB when sufficient coherence lifetime and excitation intensity are available. The color coding is used to qualitatively indicate the spectral position of the pulses.

In practice, the temporal configuration of the probe pulse introduces distinct operation regimes (Figure 2a). In many biological implementations, excitation and probing occur with minimal delay. This maximizes signal strength and allows operation at low pulse energies, which is essential to avoid photodamage in sensitive samples. The resulting strong NRB is subsequently removed using phase-retrieval or heterodyne approaches. In addition, vibrational dephasing times in dense biological media are often short, favoring near-zero operation.

In contrast, gas-phase diagnostics and studies of simple liquids frequently employ delayed probing schemes (Figure 2b). Here, vibrational coherence lifetimes are comparatively long, and higher pulse energies are applicable. Introducing a controlled probe delay suppresses the instantaneous NRB contribution and allows background-reduced detection of resonant features. The availability of μJ-level pulse energies compensates for the signal reduction associated with delayed probing.

Consequently, while both communities may formally operate within either two- or three-color excitation frameworks, the combination of spectral architecture and temporal strategy determines signal scaling, background management, and experimental constraints. The following sections discuss technical approaches for broadband generation largely independent from these temporal considerations.

### 2.1. Oscillator-Based Broadband CRS

The most direct approach of broadband CRS exploits the intrinsic spectral width of fs oscillators without external amplification or nonlinear broadening stages. Architecturally, this represents the minimal implementation of multiplex CARS: the oscillator spectrum is spectrally split or shaped to provide the required excitation field. Early fs-Ti:sapphire implementations—like the above-mentioned studies of the Silberberg group [10]—established the feasibility of intrapulse broadband excitation using the unamplified oscillator output, forming the conceptual basis of multiplex CARS microscopy.

Oscillator-driven schemes operate in the nJ pulse energy regime but at high repetition rates (typically tens of MHz), providing efficient signal averaging and rapid statistical sampling of short-lived vibrational coherences. Subsequent developments explored spectral shaping of nearly the full oscillator bandwidth. For example, Isobe et al. employed a spectrum spanning approximately $\Delta \tilde{\nu }$ ~ 4800 cm^−1^ for Fourier-transform (FT-CARS) and differential spectral focusing within the same platform. Similarly, the Motzkus group demonstrated selective excitation of vibrational mode using pulse-shaping techniques, including linear chirps to enhance the CH-stretch resonances around 2950 cm^−1^ [16,17].

In single-beam, oscillator-based implementations the emphasis is on spectral field engineering, e.g., to provide the narrowband probe pulse. In this regime, the achievable spectral performance is therefore closely linked to coherent control of excitation fields. A detailed discussion of pulse-shaping concepts in CARS is provided in a recent review and will not be repeated here [18].

However, when higher pulse energies or delayed probing schemes are required—such as in low-density gases or long-lived coherences [19,20]—external nonlinear broadening or amplification stages become necessary, as discussed in the following sections.

### 2.2. Supercontinuum Generation in Bulk Materials

An alternative route to broadband excitation is supercontinuum generation in bulk nonlinear media or solid-core fibers. In these approaches, fs-pulses, for example, from high repetition rate ytterbium (Yb)-based fiber lasers, are spectrally broadened via nonlinear propagation in solid materials such as yttrium–aluminum–garnet (YAG). The resulting near-infrared (NIR) white-light continua can span more than 3000 cm^−1^ while maintaining energy fluctuations below 1% RMS. Narrowband pump pulses are typically derived via spectral filtering, enabling CARS resolutions on the order of 10 cm^−1^.

The physical foundations of white-light continuum generation in bulk glasses and crystals date back to the seminal experiments by Alfano and Shapiro in 1970 [21]. In the fs regime, systematic studies of Brodeur and Chin clarified the role of self-phase modulation, self-focusing and bandgap scaling in condensed media [22], establishing bulk supercontinuum generation as a controllable broadband source.

Early implementations of super-continuum-based broadband CARS employed fiber-based architectures. For example, an Er:fiber laser was used to generate a supercontinuum in a germanosilicate fiber with a bandwidth of 4000 cm^−1^ for the investigation of *C. elegans* [23]. Building on such schemes, Camp et al. demonstrated broadband CARS imaging of healthy and cancerous tissue, explicitly discussing the interplay of two-color and three-color excitation mechanisms [8]. More recently, bulk-generated continua have been applied to broadband imaging and tumor tissue discrimination, illustrating the robustness of solid-state spectral broadening platforms [7].

Moreover, supercontinuum generation in tapered [24,25] or photonic crystal fibers (PCFs) [26] provides compact broadband sources in the nJ pulse energy regime, which is particularly suited for high repetition rate microscopy implementations. In a proof-of-principle experiment, wide-field CARS imaging on polymethyl methacrylate (PMMA), polystyrene and L-cystine was demonstrated with a bandwidth up to 3050 cm^−1^ [27]. Similarly, Chen et al. employed a PCF to generate a continuum covering 400–3500 cm^−1^ for the investigation of Raman modes of benzonitrile [28].

In practice, fiber-based and bulk-supercontinuum stages occupy complementary niches. PCFs and tapered fibers provide long interaction lengths, allowing substantial spectral broadening at nJ pulse energies and MHz repetition rates in compact, alignment-stable microscope platforms. Bulk media such as YAG offer a simpler free-space alternative that avoids fiber coupling and can tolerate higher average powers, though typically requiring higher peak power to achieve comparable bandwidth.

Despite their architectural differences, both approaches generally remain within the nJ pulse energy regime. While this is fully sufficient for high-repetition-rate microscope and near-zero-delay excitation schemes, it limits excitation efficiency in low-density media or in experiments requiring delayed probing of long-lived coherences. To overcome these constraints, energy scalable broadband generation techniques—most prominently gas-filled hollow-core fibers and parametric amplification—have been developed, as discussed in the following sections.

### 2.3. Hollow-Core Fibers

Few-cycle generation via gas-filled hollow-core fibers emerged in the mid-1990s to combine pulse energies ≥ 100 mJ with octave-spanning spectral bandwidths [29]. This approach uses self-phase modulation in large-mode waveguides followed by pulse compression with chirped mirrors. Early demonstrations compressed amplified Ti:sapphire pulses down to ~5 fs at a kHz repetition rate, establishing hollow-core fibers as a scalable workhorse for high-energy few-cycle pulses.

Böhle et al. report on spectral widths of Δλ > 300 nm centered at λ = 800 nm, resulting in a minimum pulse duration of 2.6 fs [30]. The shot-to-shot stability of the spectral broadening strongly depends on the gas pressure and coupling conditions, which jointly determine the nonlinear broadening dynamics [31,32]. For high pulse energies of 3.4 mJ and very short pulses (2.6 fs), Böhle et al. reported shot-to-shot fluctuations of 7.8% (RMS) for linear and 4.7% for circular polarization [30]. In a more recent study, shot-to-shot fluctuations of the spectral width were 2.5%, and 6.8% over 24 h (see Figure 4 in [33]), but pulse parameters were more relaxed (<600 μJ, 16 fs).

Regarding CRS, Roy et al. performed the first experiments in a single-beam configuration, to provide both the broadband pump/Stokes pulse as well as a narrowband probe pulse. The narrowband probe pulse was generated by means of a spatial light modulator (SLM). With this setup, N_2_ and CO_2_ could be detected [34,35]. However, the damage threshold of the SLM limited pulse energies to a couple of μJ, making quantitative measurements difficult.

Bohlin and Kliewer addressed this limitation using two beams [13]. A 45 fs, 2 mJ pulse from a Ti:sapphire laser amplifier was spectrally broadened in a hollow-core fiber, whereas the probe was provided by a high-energy Nd:YAG laser with E_pulse_ = 30 mJ and a pulse duration of 90 ps, allowing for high-resolution spectral imaging, which resolves rotational lines in CO_2_. Results, however, clearly indicate increasing fluctuations of the CARS signal for high Raman shifts (H_2_-molecule). Subsequently, their approach was successfully applied to thermometry, species detection and imaging in flames [36,37,38]. A hollow-core fiber-based setup was also employed to demonstrate CO_2_ thermometry by means of a single spectrum [39], confirming that ultrabroadband CARS for gas analysis may be used for species detection and thermometry at the same time.

Beyond their role in spectral broadening and post-pulse compression, hollow-core fibers have also enabled dispersion managed delivery of ultrashort pulses in biomedical implementations. In particular, hollow-core fiber endoscopic systems have demonstrated in vivo CARS imaging with near dispersion free transport of fs pulses through fiber catheters, offering minimally invasive measurements and even combined imaging ablation concepts [40,41]. While these approaches typically rely on multi-beam excitation rather than ultrabroadband two-beam schemes, they illustrate the versatility of hollow-core platforms for transporting high quality ultrashort pulses into challenging environments.

### 2.4. Optical Parametric Amplification

Optical parametric amplification (OPA) is a χ^(2)^ three-wave mixing process, in which a strong pump amplifies a weak signal while also generating an idler [42]. This enables high gain and broad tunability. Optical parametric chirped-pulse amplification (OPCPA) combines OPA with CPA-style stretching and recompression, which permits amplification of ultrabroadband seed pulses to high pulse energies while mitigating nonlinear phase accumulation and thermal load [43]. As a result, OPCPA has become a key route to few-cycle, high-peak power sources from the visible to mid-IR [44].

For CRS-techniques, it is essential that the OPCPA system provides a temporally synchronized and low jitter probe pulse. A straightforward solution is to derive the probe pulse from the OPCPA pump. Owing to fiber pumping, this typically results in a wavelength in the green spectral region after second harmonic generation (SHG). While this may be disadvantageous for biomedical applications due to high tissue absorption in the visible range, it offers the advantage of comparatively high Raman scattering cross-sections, which scale favorably toward short wavelengths. Furthermore, the spectral bandwidth of the OPCPA pump must be tailored to meet the spectral resolution requirements of the CARS experiment. For efficient amplification in a noncollinear optical parametric amplifier (NOPA) stage, the spectral bandwidth is typically on the order of Δλ ~ 1 nm (corresponding to a pulse duration of ~400 fs), which translates to a spectral resolution of approximately $\Delta \tilde{\nu }$ ~ 37 cm^−1^. This resolution is insufficient for high-resolution Raman measurements and therefore often necessitates a subsequent 4f grating setup with a slit to reduce the bandwidth to the desired resolution, which comes with substantial pulse energy loss. A widely used alternative is second harmonic bandwidth compression (SHBC), which generates narrowband picosecond probes with improved energy throughput and has been demonstrated for hybrid/ps CARS thermometry [45].

In broadband OPCPA architectures, however, additional practical considerations arise. Spatio-spectral couplings such as angular dispersion and spatial chirp can be introduced during parametric amplification, particularly in noncollinear geometries [46]. These effects may remain unnoticed in spatially integrated spectral measurements but can lead, e.g., to varying excitation efficiencies for different Raman shifts. Furthermore, stable OPA operation requires precise synchronization of pump and seed pulse, as timing jitter directly affects parametric gain and, consequently, output stability [47]. When effectively managed, modern fiber-pumped OPCPA systems combine broadband amplification and synchronized dual outputs, making them attractive excitation platforms for advanced CRS applications.

As discussed above, hollow-core fiber post-compression has enabled broadband, high pulse energy excitation for demanding CRS applications. However, these Ti:sapphire-based systems are typically limited to repetition rates of about 1 kHz, which constrains temporal resolution and statistical sampling. Fiber-pumped OPCPA architectures now combine comparable pulse energies with freely adjustable repetition rates extending into the multi-10 kHz regime. For CRS, this offers two complementary advantages: improved temporal resolution for dynamic measurements and enhanced statistical averaging under fluctuating experimental conditions.

The transfer of broadband OPCPA technology into the CRS domain has progressed from custom-built systems toward commercially available fiber-pumped architectures with improved stability and tunability. These developments have enabled high-repetition-rate ultrabroadband excitation as well as adjustable probe pulse durations to spectroscopy requirements, facilitating temperature and concentration measurements in gas-phase environments and chemically reactive systems.

An early high-energy ultrabroadband CARS implementation based on a custom-built OPCPA system demonstrated repetition rates in the multi-10 kHz regime with pulse energies in the few μJ range [48]. While enabling broadband excitation, the short probe pulse duration led to spectral beating effects, for example, in the closely lying vibrational features such as the Fermi dyad of CO_2_. Subsequent developments employed commercially available fiber-pumped OPCPA platforms with adjustable probe pulse durations, allowing temperature and concentration measurements in gas mixtures over extended Raman shifts, including H_2_ [49]. The setup was further applied to chemically reactive systems and gas conversion processes [50].

Although the number of OPCPA-based CRS implementations remains comparatively limited, the rapid maturation of fiber-pumped parametric platforms suggests increasing convergence between ultrafast laser development and nonlinear spectroscopic applications. For example, the same source concept has also been extended to liquid-phase spectroscopy, demonstrating ultrabroadband CARS of complex organic systems and highlighting the versatility of energy-scalable OPCPA platforms for rapid analysis in chemically diverse environments [51].

### 2.5. Filamentation

A conceptually distinct approach to broadband excitation is filamentation, in which spectral broadening is generated directly along the propagation path rather than in a dedicated source stage. While hollow-core fibers, bulk media, and parametric amplification generate bandwidth in a dedicated source stage, filamentation produces broadband radiation along the propagation path. This leads to significant relaxation of the laser requirements and physics driven constraints.

Filamentation arises from a dynamic balance between Kerr lens self-focusing and plasma generation [52]. For broadband CRS, its most important feature is substantial spectral broadening, which for Ti:sapphire seed pulses centered at l = 800 nm can extend from the UV well into the NIR. Beyond spectral broadening, filamentation offers characteristics particularly attractive for remote sensing: (i) self-compression, where nonlinear spectral broadening combined with anomalous dispersion shortens the pulse during propagation—in some cases approaching the few-cycle regime without external compensation [53], (ii) robustness against adverse conditions such as pressure variations or turbulence [54,55], and (iii) beam propagation beyond the diffraction limit up to the kilometer scale [56]. Moreover, the onset of filamentation can be positioned at a defined location by a suitable choice of the focusing geometry and an appropriate negative pre-chirp.

A fundamental study of filamentation-based CARS was performed by Odhner et al. in 2009 [57], using a 35 fs pulse from a Ti:sapphire amplifier to generate extended air filaments. The second harmonic at λ = 400 nm served as a probe. Efficient spectral broadening enabled the detection of O_2_, N_2_ and H_2_ and up to at $\Delta \tilde{\nu }$ ~ 4200 cm^−1^. In addition, proof-of-principle thermometry at room temperature was demonstrated via analysis of the Raman coherence decay.

Filamentation-based CRS was later revisited by Mazza et al. for combustion diagnostics [58,59]. Thermometry of CO_2_, O_2_, and CH_4_ was successfully performed, including measurements in a closed reactor, where the filament was generated behind a BK7 window [60]. Sufficient transmission without damage was achieved by external pulse compression and negative chirp control. Although earlier studies demonstrated Raman shifts up to $\Delta \tilde{\nu }$ ~ 4200 cm^−1^, subsequent flame measurements were typically limited to $D\tilde{\nu }$ < 2000 cm^−1^ to maintain acceptable SNRs.

Alternatives to Ti:sapphire systems have also been explored. While mobile platforms such as Teramobile have demonstrated operation under adverse outdoor conditions [61], oscillator stability can remain sensitive to environmental fluctuations. Fiber-based systems, particularly Yb-based thin-disk architectures operating at λ = 1030 nm, offer improved robustness. Houard et al. reported filamentation using a high-power, Yb thin-disk system; however, pulse durations of ~1 ps are substantially longer than for Ti:sapphire systems, reducing the initial bandwidth prior to nonlinear broadening. Zhao et al. used IR filamentation in air with pulse energies up to 45 mJ to detect O_2_, N_2_ and CH_4_ ($\Delta \tilde{\nu }$ ~ 3000 cm^−1^) [62], though further bandwidth optimization is required for acceptable SNR for higher Raman shifts.

In this context, air lasing represents a promising probe mechanism. Air lasing refers to strong, narrowband emission generated within the filament at wavelengths such as λ = 337 nm, 358 nm, 391 nm or 428 nm [63]. Because the emission is temporally and spatially co-aligned with the filament and exhibits narrow spectral width (Δλ ~ 0.3 nm ≜ ~ 1 ps) [64], it can act as a naturally synchronized probe. Zhao et al. demonstrated proof-of-principle CARS detection of N_2_, O_2_, CO_2_ and CH_4_ using air lasing at λ = 391 nm, though with significant NRB [65]. Subsequent studies improved SNR, for example, detecting SF_6_ at 0.38% concentration using air lasing at 337 nm [66] and further improving detection limits via additional seeding at 400 nm [67]. Air lasing at λ = 428 nm was used as a probe to detect O_3_ and NO_2_ generated during filamentation itself [68]. More recently, hybrid concepts extended filament supercontinuum generation into the deep UV using internally generated 266 nm radiation in combination with 400 nm excitation [69]. Finally, Lu et al. demonstrated single-shot, single-beam thermometry on O_2_ via rotational coherent Stokes Raman scattering (CSRS) by employing a two-step configuration in which N_2_^+^ lasing was generated in a separate cell prior to interaction with O_2_ [70].

In summary, filamentation offers a conceptually distinct approach in which broadband generation, self-compression, and long-distance propagation are intrinsically coupled. This makes it particularly attractive for applications in harsh or confined environments, such as industrial reactors, or combustion chambers, where robustness and in situ bandwidth generation are advantageous. At the same time, many implementations rely on auxiliary probe beams or multi-stage configurations, which increases experimental complexity. Air-lasing concepts offer a promising route toward more self-contained schemes. Overall, filamentation occupies a unique position among broadband CRS strategies, combining high pulse energy scalability with environmental robustness, albeit within a less modular system architecture than source-based approaches.

While increasing bandwidth is a prerequisite for multiplex detection, it also introduces additional challenges for signal interpretation. In particular, if broadband excitation is not properly compressed or phase-controlled, the excitation efficiency can vary significantly across the spectral range. As a result, the measured CARS spectra no longer reflect the molecular response but are modulated by the spectral structure of the excitation field. Moreover, broader bandwidths inherently increase the complexity of the interference between resonant and non-resonant contributions. This leads to more structured and sample-dependent backgrounds, complicating quantitative analysis and spectral retrieval. Consequently, broadband excitation enhances multiplex capability, but at the same time increases the demands on phase control, calibration, and data processing.

## 3. Detection and Spectral Retrieval Challenges

With the advent of broadband excitation schemes, the CRS community gained access to increasingly multiplex vibrational information. At the same time, this expansion in spectral bandwidth introduced new challenges in signal detection and spectral retrieval. As described by

(3) $$\chi^{\left(3\right)}=\chi_{NR}^{\left(3\right)}+\chi_{R}^{\left(3\right)},$$

the detected CARS signal arises from the coherent superposition of resonant and non-resonant contributions. With increasing bandwidth, many Raman shifts are driven simultaneously, and the interference between $\chi_{R}^{\left(3\right)}$ and $\chi_{NR}^{\left(3\right)}$ becomes correspondingly more intricate. Broadband excitation therefore enhances multiplex capability, but at the same time increases the dimensionality of the interference problem.

**Figure 3:**
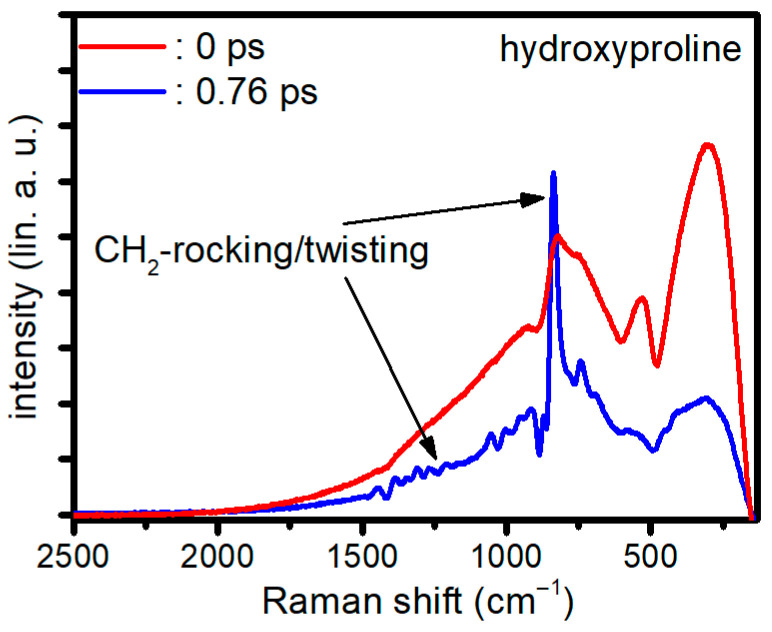
CARS spectra of 25% concentrated hydroxyproline in water taken for a probe pulse delay of 0 fs and 760 fs respectively. The measurements are the accumulation of 100 spectra, each with 50 ms exposure duration.

To illustrate this behavior, Figure 3 displays the CARS spectra of hydroxyproline dissolved in water at a 25% concentration at different time delays. The experimental conditions are comparable to those reported in Ref. [51]; however, the probe delay is chosen differently here to more clearly illustrate the temporal evolution of the signal. When delaying the probe by its own duration, in this case 760 fs, the NRB is effectively suppressed, albeit at the expense of reduced resonant signal intensity. Nonetheless, the peaks of the CH_2_ rocking and twisting band cover the fingerprint region around 500–1600 cm^−1^, with the most eye-catching peak being the CH_2_ twisting at 844 cm^−1^.

This example highlights the central detection challenge of broadband CRS: maximizing vibrational specificity while managing the non-resonant background. To remove the NRB, several approaches have been developed in the biomedical broadband CARS field. They can be distinguished into experimental setup-based, algorithmic phase retrieval and deep learning (DL)-based phase retrieval. An overview of these approaches and their applications is given in the next sections. In the following section, we adopt the common biomedical term BCARS for broadband CARS.

### 3.1. NRB Removal—Experimental Approaches

The BCARS signal of biological samples tends to be complex, as many different and intricate molecules create overlapping signals of low intensity. Solutions such as increasing the laser power are limited by the fragility of the sample. Furthermore, the intrinsic NRB covers most of the resonant peaks. Therefore, developing different methods of NRB removal and reduction have been a priority in the field.

The first steps taken were experimental configuration-based approaches. Some of them are time-resolved CARS, polarization CARS, heterodyne CARS or phase-shaping CARS. As described in Section 2, time-resolved CARS works based on the underlying temporal mechanics of the resonant and non-resonant components of CARS. FAST-CARS is a newer approach where, additional to the probe delay, the pulse duration is adjusted to improve the resonant signal intensity while keeping the NRB suppression and spectral resolution high [71]. Similarly, other methods use other features of CARS such as the polarization of signals. These are further described in the review paper by Junjuri et al. [72]. For all of them, a removal of or significant reduction in background was shown. However, they also generally tend to decrease the signal intensity and prove sensitive to misalignments or phase instabilities. The complex setups also come with higher costs [72]. Consequently, these methods are unsuited for current in-field applications but might prove advantageous in static laboratory conditions for basic research. Varying types of samples and experimental conditions may need to be considered in basic research when testing processes around and in tissue, such as ablation or photo-chemical reactions. For those, a combination of experimental setup adaptations with established tactics such as algorithm and DL-based phase retrieval can be considered.

### 3.2. Algorithmic Phase Retrieval

Preferred and established for in-clinic studies are phase retrieval methods such as the Kramers–Kronig (KK) algorithm, maximum entropy method (MEM) or Hilbert transform. A major step forward in this field was made by the group around Camp. In 2014 they used heterodyne amplification to enhance the resonant signal in their measurements while removing the remainder of NRB with the KK algorithm. A co-seeded fiber laser supplied a narrowband ps and a broadband fs output. Their probed BCARS spectrum ranged over 500–3500 cm^−1^, making use of both the “2-colour” scheme for OH/CH-vibration excitation and “3-colour” CARS to excite the fingerprint region. Along with two-photon autofluorescence and SHG, the highly detailed spectral maps across 400 μm^2^ sized areas offered a vast multitude of opportunities to color code images and differentiate tissue types. At that point, however, automizing tissue identification was still lacking the needed data banks [7]. In 2023 Vernuccio et al. used a similar approach, extracting information from SHG and CARS at 400–3400 cm^−1^ using the time-domain KK algorithm. Simultaneously to the spectra acquisition, their high-speed post-processing immediately identified lipids, proteins and nuclei. An 800 μm × 800 μm area was scanned at 3 ms pixel dwell time, summing up to a total of just 8 min imaging time. The applied energy was kept at 17.5 nJ. This approach enabled tumor imaging with a quality comparable to the medical gold standard H&E staining, while being less invasive and significantly faster [7]. A very recent study by Ebersbach et al. (2025) [73] built yet again on a setup based on the work of Camp et al. [8], but made use of the Hilbert transform to remove the NRB. They only focused on the most important spectral ranges of 600–1800 cm^−1^ and 2800–3100 cm^−1^. The covered volume of 193 μm × 200 μm × 30 μm was scanned slower than in previous studies, at 10 ms per pixel. Nonetheless, in fewer than 30 min a high-resolution scan of plant leaves was created. The higher quality spectra opened opportunities for precise analyses. After several complex post-processing steps, a total of 10–20 endmembers were defined. The color-coded images gave detailed insight into the chemical compositions and cell structures of leaves in sub-cellular resolution.

The reliability of these algorithms has made them widely adopted in practice. However, despite providing high-quality spectral reconstruction, algorithmic phase retrieval typically requires a reference spectrum of the NRB. The reconstruction process can introduce phase and amplitude errors, which often necessitate additional correction steps and may limit the achievable accuracy. Furthermore, adapting these methods to specific experimental setups can be nontrivial and may involve user-dependent choices, potentially affecting the objectivity of the results.

Deep learning approaches aim to overcome these limitations by eliminating the need for explicit NRB reference measurements, reducing the reliance on subsequent correction procedures, and minimizing user intervention.

### 3.3. Deep Learning Approaches

DL only evolved in recent years to bring forth different phase retrieval systems. In 2020 a convolutional neural network (CNN) named SpecNet was first trained to retrieve Raman spectra from the NRB [74]. A total of 30,000 simulated spectra served as a training databank. In the same year a long short-term memory (LSTM) network was trained with 4000 simulated spectra [75]. Both methods proved to take only a few milliseconds, being in the same range as typical BCARS spectral acquisition times and algorithmic phase retrieval. Consequently, they may be used for real time imaging during the scanning process. Applied to different fluids they presented Raman-like results with root mean square errors lower by up to one power of magnitude than KK and MEM. However, when tested on more complex spectra like tissue signals, the results lost their accuracy [74].

In 2022 Wang et al. [76] released the Very Deep Convolutional Autoencoders (VECTOR) model. This model used a total of nine datasets, each with 50,000 simulated spectra, for training. The model demonstrated robust performance even for complex and heterogenous samples, making it applicable to tissue measurements. However, earlier approaches such as SpecNet, LSTM, and the aforementioned VECTOR generally exhibit limitations in accurately reconstructing low-intensity spectral features and the edges of recorded spectra. To address these challenges, Junjuri et al. developed a Bi-LSTM model in 2023, which showed better agreement with spontaneous Raman scattering (SpRS) spectra, particularly for weak spectral features, albeit at the cost of increased post-processing time [77]. More recently, Vernuccio et al. (2024) [78] introduced approaches based on generative adversarial networks (GANs) and CNN with gated recurrent units, achieving enhanced spectral accuracy and improved detection of low-intensity peaks while maintaining short processing times. Among these, the GAN-based method demonstrated the highest peak detection performance combined with minimal computational cost.

However, the performance of DL models strongly depends on the availability and quality of the training data. Large and representative datasets are required to ensure reliable performance across different samples and experimental conditions [72]. These datasets can be obtained either experimentally or through simulation. In practice, simulated datasets are often preferred due to the time-consuming nature of acquiring training data under realistic conditions. It is therefore crucial that simulated data accurately reflect experimental conditions, including noise levels, signal amplitudes, and spectral variability. Mismatches between training and experimental data can lead to reduced performance in practical applications. Consequently, the development of comprehensive and representative training datasets remains a key challenge.

Overall, the current state of the art in DL-based phase retrieval is highly promising, and further integration into real-time tissue imaging workflows appears to be a natural next step. These datasets must reflect variations in measurement conditions and tissue composition. Training data can be obtained either experimentally or through simulation. However, acquiring sufficiently large datasets under realistic (e.g., clinical) conditions is often time-consuming, making simulation the more practical approach.

It is therefore essential that simulated data closely reproduce experimental conditions, including realistic noise levels, signal amplitudes, and spectral variability. Systematic discrepancies—such as underestimated noise or overestimated resonant signal strength and stability—can lead to reduced performance in practical applications. Consequently, the development of more comprehensive and representative training datasets, as well as ongoing model retraining, remains a key priority [78].

### 3.4. Speed, Processing Time and Practical Constraints

Multiple factors must be considered in CARS measurements of biological tissue, as they directly impact both experimental design and achievable performance. Key parameters include acquisition time, SNR, spectral bandwidth and resolutions, permissible excitation energy, and the stability of measurement conditions, such as sample properties and phase-matching requirements.

Acquisition time is a critical parameter, particularly for volumetric tissue imaging. However, biological samples typically exhibit low signal levels and are sensitive to photodamage, with ablation thresholds of a few J/cm^2^. This imposes constraints on excitation energy and often necessitates longer integration times, the use of highly sensitive detectors, and efficient data processing strategies.

Charge-coupled device (CCD)-based detection systems offer high sensitivity combined with acquisition times in the ms range, making them well suited for many imaging applications. Nevertheless, efforts to further increase acquisition speed have been reported. For example, Kizawa et al. [79] employed an elliptical focus to scan living cells, enabling the acquisition of up to 34,100 spectra per second over a spectral range of 900 cm^−1^. In this work, non-resonant background removal was performed using the maximum entropy method (MEM). However, despite the high acquisition speed (0.1 µs per pixel), post-processing remained a bottleneck, requiring approximately 10 min per image due to the large number of spectra per frame.

Algorithmic and DL-based phase retrieval can be performed on timescales comparable to data acquisition, often operating in parallel with spectral recording. However, post-processing of CARS data extends beyond phase retrieval alone. A complete processing pipeline typically comprises three major steps: (i) data denoising, (ii) NRB removal including phase and amplitude correction, and (ii) spectral unmixing. Denoising is essential for improving the SNR. Various approaches have been proposed, including spectral total variation, originally introduced for stimulated Raman scattering (SRS) and shown to improve the SNR by up to a factor of 57 [80], as well as singular value decomposition (SVD), which is widely used in BCARS data processing [7,8,81].

In 2020, Camp et al. [82] introduced the factorized Kramers–Kronig and error correction (fKK-EC) approach, which combines SVD, KK phase retrieval, and phase and amplitude correction into a unified framework. This method significantly reduces computational effort, enabling processing times of approximately 0.1 ms per spectrum—about an order of magnitude faster than typical acquisition times. However, similar to machine learning approaches, fkk-EC relies on representative training data for optimal performance.

In recent years, machine learning techniques have also been applied directly to both denoising and NRB removal [83,84,85,86]. While these methods can significantly reduce processing time, they may introduce artifacts such as spectral blurring or overfitting, potentially limiting the reliability of quantitative analysis. This is particularly critical for multiplex spectra, where accurate identification and quantification of multiple spectral components is required. As with phase retrieval, the performance of DL-based denoising depends strongly on the representativeness of the training data. Consequently, such approaches are currently more robust for narrowband CARS or SRS applications, where spectra complexity is reduced.

Overall, the state of the art in BCARS imaging has progressed to a level where spectra can be acquired and processed on ms timescales without significant sample damage. The label-free and non-invasive nature of the technique offers clear advantages over conventional staining methods. Combined with high spatial resolution, BCARS enables subcellular imaging and the detection of subtle compositional changes in tissue [87]. This has been demonstrated, for example, in the identification of tumor margins and differentiation between healthy and cancerous tissue [7], and holds promise for monitoring disease progression and treatment response in fields such as oncology and neurodegeneration [88,89,90,91,92].

Despite these advances, achieving simultaneously high acquisition speed, broad spectral coverage and robust data processing remains a key challenge. As demonstrated by Kizawa et al., acquisition rates of up to ~34,100 spectra per s can be achieved using an elliptical focus; however, post-processing can become the limiting factor, requiring up to 10 min per image due to the large data volume [79]. Future progress will therefore rely on the combined development of faster detection schemes, more efficiently processing algorithms, and optimized excitation strategies. In particular, approaches that distribute excitation energy over larger sample areas while maintaining control over photodamage may enable further increases in imaging speed.

To place broadband CRS in context, it is instructive to compare its performance with related vibrational spectroscopy techniques. SpRS provides high spectral fidelity and straightforward interpretation, but suffers from low signal levels, typically requiring long acquisition times (e.g., a few s) or high excitation intensities. In contrast, CRS techniques offer signal enhancements of several orders of magnitude (typically 10^4^–10^8^ compared to SpRS), enabling faster acquisition and improved sensitivity.

Narrowband CARS—still widely used in biomedical applications—covers only limited spectral regions, reducing interference between resonant and non-resonant contributions and simplifying post-processing and spectral unmixing. Typical applications include imaging of tissue morphology, such as tumor detection or skin analysis, as well as studies of transport processes like drug or water penetration [93,94,95,96,97]. The multiplex capability is lost in this approach.

Broadband SRS is—in contrast to CARS—free from NRB, but typically requires spectral scanning or more complex detection schemes to achieve comparable bandwidth coverage. Nevertheless, a total bandwidth of 400–3600 cm^−1^ has been demonstrated recently, with a resolution $\Delta \tilde{\nu }$ ~ 10 cm^−1^ [98], using a combination of two spectral scanning techniques.

Frequency-comb CRS approaches, such as dual-comb Raman spectroscopy [99], enable broadband spectral acquisition with high resolution and precise frequency calibration. However, these systems often involve increased experimental complexity and may be limited in pulse energy, for example, when relying on two independent Ti:sapphire oscillators.

Overall, broadband CRS occupies a regime that combines high signal levels, wide spectral coverage, and flexible temporal resolution. A potential setup providing these options, as well as first application testing, are introduced in Section 4.

## 4. A Scalable Ultrabroadband Platform for Advanced CRS Applications

Advanced CRS schemes require excitation sources that combine broad spectral coverage with high pulse energies and excellent shot-to-shot stability. From the considerations discussed above, parametric amplification represents a particularly suitable approach to meet these requirements.

**Figure 4:**
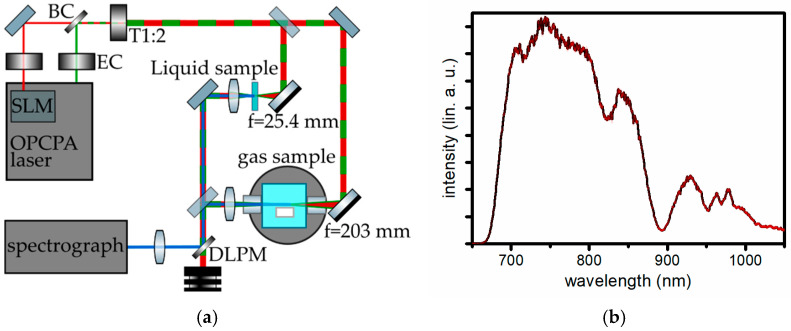
(**a**) Schematic of ultrabroadband CARS setup. SLM—spatial light modulator, BC—beam combiner, EC—energy control, T1:2—beam expander 1:2, DLPM—dichroic long-pass mirror; (**b**) single-shot spectrum of the pump/Stokes pulse (black line) and its shot-to-shot standard deviation over 50 measurements (red line).

To illustrate the versatility of OPCPA-based excitation for ultrabroadband CRS, we present representative measurements with a custom-built dual-output OPCPA system (ORPHEUS-OPCPA, Light Conversion, Vilnius, Lithuania) (Figure 4a). The main output delivers near-transform-limited pulses of ~7 fs with a highly stable spectral shape which spans more than 5000 cm^−1^ (690–1080 nm) (Figure 4b). Pulse compression is achieved via multiple reflections on custom chirped mirrors (Light Conversion), providing tailored negative second- and third-order dispersion. Maximum pulse energies are 100 µJ at repetition rates freely adjustable between 1 Hz and 100 kHz. The laser system is passively carrier-envelope phase (CEP)-stabilized, ensuring excellent shot-to-shot reproducibility. Optionally, a spatial light modulator can be inserted into the beam path to allow for arbitrary phase manipulations. A secondary output, derived from the pump laser and frequency converted in a second harmonic bandwidth compressor (SHBC, Light Conversion, Lithuania), provides a spectrally narrow probe pulse centered at 513 nm with a bandwidth of 7.7 cm^−1^, pulse energies up to 32 µJ, and an adjustable pulse duration, currently set to ~2.6 ps. An internal delay stage ensures precise temporal synchronization of excitation and probe pulses.

Both beams are equipped with individual wave plates and polarizing beam splitters for continuous pulse energy control. Spatial combination is performed by a dichroic mirror. A reflective Galilean telescope expands the beam diameter to ~10 mm prior to focusing.

Gas analysis is performed in a high-temperature, high-pressure gas reactor (Linseis Messgeräte GmbH, Germany) equipped with optical access ports. The pump/Stokes and probe pulses are focused into the reactor using an off-axis parabolic mirror (f = 203 mm). A negative pre-chirp is applied to the broadband excitation pulse to compensate for dispersion introduced by the reactor windows. The CARS signal is collected in transmission using a collimating lens. For liquid samples, both pulses are collinearly focused using a short focal length and off-axis parabolic mirror (f = 25.4 mm), and the collected CARS signal is likewise collected by a collimating lens.

The CARS signal is spectrally separated from the excitation beams using cascaded optical filters and analyzed by a Czerny–Turner spectrometer (Shamrock 500i, Oxford Instruments, High Wycombe, Buckinghamshire, UK) equipped with a triggered, cooled CCD camera (minimum exposure time 4.5 ms).

The high pulse energies available from the OPCPA system enable excitation over large interaction volumes and provide high single-shot signal levels. To illustrate this, exemplary measurements are shown below. All spectra are captured without algorithmic NRB removal. A temporal probe pulse delay is used to suppress the NRB to only detect resonant molecular information in the CARS spectra. The adaptable repetition rate of the laser system gives us the possibility to observe single-shot measurements with standard gated scientific cameras.

**Figure 5:**
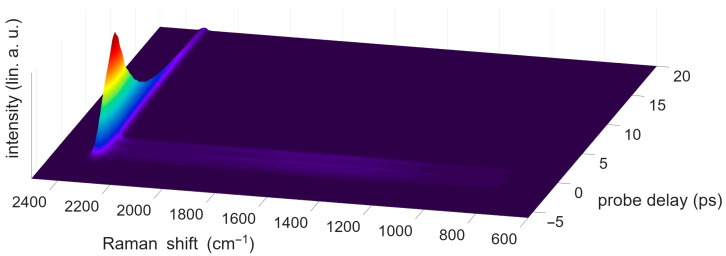
Single-shot CARS signal of N_2_ gas, with p_Sample_ = 2.0 bar. The probe delay is scanned from –6 ps to 20 ps. The colors represent intensity, with red indicating high and blue low values.

First, the evolution of the CRS signal of N_2_ gas over an increasing probe pulse delay is shown in Figure 5. To allow the acquisition at different probe delay times, a delay stage was moved in increments of 0.33 ps. The zero-delay position was determined as the maximum of the NRB in pure argon. The temporal shape of the NRB matches the temporal shape of the probe pulse. In Figure 5, the NRB is visible as a broadband signal of low intensity around the zero-probe-delay line. All molecular information is carried in the resonant CRS signal at 2330 cm^−1^. The decay time of this signal matches the observed decay time from [100], which is much longer than that of the NRB.

**Figure 6:**
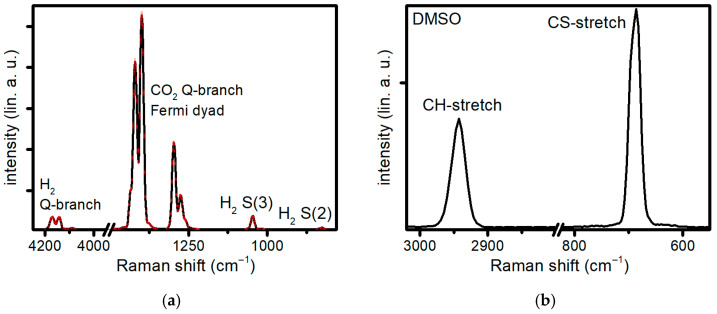
(**a**) Single-shot CARS signal of a 50:50 gas mixture of H_2_ and CO_2_, with p_Sample_ = 2.0 bar and T_Sample_ = 600 °C. The probe delay was set to 2.66 ps, right after the full decay of the NRB. Signal standard deviation over 50 single-shot measurements is indicated as a red envelope around the spectrum. (**b**) Single-shot CARS signal of DMSO. Pulse energies of E_pump/stokes_ = 250 nJ and E_probe_ = 350 nJ were used. Note that the axis break is used solely to improve the visibility of the spectral peaks; the single-shot spectra were acquired over the full wavenumber range.

An additional experiment in the gas phase is shown in Figure 6a to express the possibility of performing thermometry as well as simultaneous multiplexed gas diagnosis. Here, a 50:50 mixture of H_2_ and CO_2_, heated to 600 °C, is detected inside the gas reaction chamber. Low pulse energies of E_pump/Stokes_ = 2 µJ and E_probe_ = 1 µJ are used to prevent detector saturation. The vibrational transitions corresponding to both gas species are identified. By comparing 50 individual spectra, the standard deviation of each data point was retrieved and is shown as an envelope of the spectrum. The low fluctuation level demonstrates that the high stability of the laser system translates directly into a highly reproducible CARS signal. This reproducibility is primarily governed by the stability of the ultrabroadband pump/Stokes pulse. As a result, even weak high-frequency features, such as the vibrational H_2_ Q-branch, can be reliably resolved, as shown in Figure 6a. Thermometric analysis of gases with CARS is done by probing the population of excited vibrational levels. The population of these levels is directly correlated to the thermal energy of the molecule, and thus gives thermometric insight [39]. The elevated population of these vibrational states gives rise to sidebands from the initial Raman transitions, so-called Fermi-Dyads. These Fermi-Dyads can be seen in the measured spectra for CO_2_ and H_2_. The multiplex detection, as well as thermometric analysis of gases in harsh environments, also offers potential for in situ investigations of gas conversion processes and analysis of organic fluids and potentially tissue samples. Here, a single-shot CARS spectrum of DMSO is shown in Figure 6b. The characteristic peak in the fingerprint region at 670 cm^−1^ as well as the CH-stretching peak at 2913 cm^−1^ are clearly identified, consistent with the multiplexing approach. To prevent sample destruction and thermal effects, pulse energies far below 1µJ are used and the repetition rate is reduced to 10–1 Hz. Both experiments are performed with only fractions of the total available pulse energy, giving a large headspace for future power scaling or modifications to benefit the analysis or possible NRB removal technologies.

Comparing the peak ratios, it becomes evident that the excitation efficiency varies across the spectral range. This can be attributed, in part, to the different Raman cross-sections of the individual transitions. In addition, the number of contributing pump/Stokes frequency pairs within the ultrabroadband spectrum depends on the Raman shift Ω, further modulating the excitation efficiency.

As introduced in Section 2, the spectral driving term A(Ω) can be interpreted as the effective excitation efficiency of the ultrabroadband pulse. It represents the coherent sum over all pump/Stokes frequency pairs separated by Ω within the excitation bandwidth. In the absence of molecular resonances, the convolution of this term with the probe field yields a purely non-resonant CARS signal.

By measuring such a non-resonant signal in a noble gas (e.g., argon) and accounting for the probe field, the excitation efficiency can be experimentally retrieved. This approach has been demonstrated previously and allows the derivation of a correction factor directly from the non-resonant spectrum [38].

These observations highlight that broadband excitation introduces additional complexity in the excitation process, which merits further investigation. A promising approach is the selective control of the spectral amplitude and phase of the pump/Stokes pulse.

Using an SLM, the effective excitation term A(Ω) (Equation (2)) can be tailored by selectively enhancing or suppressing specific spectral components. In this way, the number of contributing pump/Stokes frequency pairs for a given Raman shift can be controlled, effectively reducing the broadband excitation to a more well-defined subset of interactions. In the limiting case, it approaches the behavior of a narrowband CARS configuration, where the excitation pathways are well defined.

Such control can be implemented through different strategies. For example, phase shaping (e.g., quadratic profiles) can be used to preferentially enhance specific vibrational transitions, as demonstrated in previous work [16]. More generally, adaptive optimization schemes, such as genetic algorithms, may be employed to maximize the signal of selected CARS features. In addition, controlled variation of linear chirp provides a well-defined means to investigate the CARS excitation process. For few-cycle pulses (~7 fs), even minor residual dispersion can substantially modify the temporal field, making precise control of the spectral phase essential. Implementing chirp control via a programmable SLM therefore enables systematic studies under well-controlled conditions. In contrast to introducing dispersion through additional transmissive optics, this approach avoids changes in the beam path and alignment, and parasitic effects such as Fresnel reflections.

By comparing controlled excitation schemes with the intrinsic ultrabroadband response, further insight into excitation efficiency and its spectral dependance can be obtained. To enable such studies, a supplementary SLM has been integrated into our laser system for future measurements.

The presented measurements illustrate that broadband, energy-scalable OPCPA excitation opens regimes that were previously difficult to access with a single platform. The availability of stable single-shot spectra enables thermometry, multiplex species detection, and vibrational fingerprinting without the need for extensive averaging. In liquid-phase samples, stable single-shot acquisition reduces cumulative thermal load, which may be particularly relevant for sensitive organic systems or biological environments.

At the same time, the combination of high pulse energy and an SLM provides a route toward controlled excitation strategies beyond passive multiplex detection. In analogy to low-energy coherent control approaches, selective enhancement of specific vibrational pathways through tailored pump/Stokes phase profiles becomes feasible at substantially higher pulse energies. This may allow targeted amplification of weak transitions, suppression of competing pathways, or adaptive optimization for complex gas mixtures.

Finally, the adjustable repetition rate up to 100 kHz provides a bridge toward the investigation of highly dynamic processes. Modern gated detection schemes and high-speed cameras can match these repetition rates; the remaining challenge shifts from temporal resolution to data throughput and real-time processing of large spectral datasets. In this regime, broadband CRS may move beyond static characterization toward time-resolved monitoring of transient gas-phase reactions, catalytic conversion, or electrochemical systems under operation. The ability to probe gases and liquids with the same setup additionally opens perspectives for studying phase transitions and multiphase processes. While aspects such as optical access, beam propagation in heterogeneous environments, and signal stability in multiphase systems still require careful experimental optimization, the combination of single-shot sensitivity and high repetition rate sampling provides a promising route toward application-driven, operando vibrational diagnostics.

## 5. Conclusions

Broadband CRS has evolved into a versatile toolbox of excitation architectures, ranging from oscillator-based intrapulse schemes to supercontinuum generation, hollow-core compression, parametric amplification, and filamentation. Although these approaches differ in pulse energy, repetition rate, and implementation, they address the same fundamental objective: accessing extended Raman bandwidths within a single acquisition.

The divergence between gas-phase diagnostics and biomedical spectroscopy reflects differing physical constraints rather than incompatible concepts. Ultimately, bandwidth, coherence lifetime and excitation intensity define the operational regime. As ultrabroadband sources become more stable, scalable and flexible, and as detection and retrieval strategies mature, these regimes are beginning to converge.

The presented OPCPA-based implementation illustrates this convergence by providing a representative platform that highlights accessible operating windows, single-shot stability, and multiplex detection under realistic experimental conditions. While OPCPA-based platforms currently represent a technologically demanding approach, they provide a unique opportunity to explore regimes that are not accessible with more conventional laser systems. In particular, the combination of high pulse energy, ultrabroad bandwidth, and precise temporal control enables the investigation of highly dynamic processes, such as transient species formation, plasma-assisted reactions, or photo-induced processes in liquids. Beyond their immediate application, such systems serve as exploratory platforms to identify the relevant observable, timescale and spectral requirements for specific problems, thereby guiding the design of simplified and more application-specific CRS implementations. For example, in photocatalytic reaction studies, where the relevant reaction pathways, reaction intermediates and product species may not be known a priori, broadband excitation allows simultaneous detection across a wide spectral range. Once the key species and spectral regions are identified, more targeted and less complex excitation schemes can be developed for routine measurements.

Looking ahead, further progress in broadband CRS is likely to be driven by three interconnected directions: (i) the development of more compact and stable broadband laser sources that retain key performance parameters while reducing system complexity, (ii) advances in data processing and real-time spectral retrieval to fully exploit multiplex information under realistic conditions, and (iii) the translation of high-end platform insights into cost-efficient, application-tailored excitation schemes. Together, these developments are expected to enable a transition from specialized laboratory systems toward more robust and widely accessible CRS technologies.

## Data Availability

No new data were created or analyzed in this study. Data sharing is not applicable to this article.

## Abbreviations

The following abbreviations are used in this manuscript:

BCARS	Broadband coherent anti-Stokes Raman scattering
CARS	Coherent anti-Stokes Raman scattering
CCD	Charge-coupled device
CEP	Carrier-envelope phase
CNN	Convolutional neural network
CRS	Coherent Raman scattering
CSRS	Coherent Stokes Raman scattering
DL	Deep learning
FAST-CARS	Fs adaptive spectroscopic techniques for CARS
FT-CARS	Fourier-transform CARS
GAN	Generative adversarial network
KK	Kramers–Kronig
LSTM	Long short-term memory
MEM	Maximum entropy method
NIR	Near-infrared
NOPA	Noncollinear optical parametric amplifier
NRB	Non-resonant background
OPA	Optical parametric amplification
OPCPA	Optical parametric chirped pulse amplification
PCF	Photonics crystal fiber
PMMA	Polymethyl methacrylate
RMS	Root mean square
SHG	Second harmonic generation
SHBC	Second harmonic bandwidth compression
SLM	Spatial light modulator
SNR	Signal-to-noise ratio
SpRS	Spontaneous Raman scattering
SRS	Stimulated Raman scattering
VECTOR	Very Deep Convolutional Autoencoders
YAG	Yttrium–aluminum–garnet
Yb	Ytterbium
